# Investigation on Occupational Protection and Exposure of Medical Staff in the Diagnosis and Treatment of COVID-19 in Sichuan

**DOI:** 10.1017/ash.2021.100

**Published:** 2021-07-29

**Authors:** Wenzhi Huang, Zhiyong Zong, Fu Qiao, Ji Lin

## Abstract

**Background:** We investigated the contact status of medical staff with confirmed or suspected patients with COVID-19 in Sichuan Province, China, as well as the use of personal protective equipment at the time of contact, and we explored the factors affecting the effective use of personal protective equipment. **Methods:** We performed a cross-sectional study by distributing a questionnaire on occupational protection and exposure of medical staff in the diagnosis and treatment of COVID-19 using a convenience sampling method for online surveys from February 23 to February 29, 2020. **Results:** In total, 13,829 valid questionnaires from 644 hospitals in Sichuan Province were retrieved, and 802 people were exposed to confirmed or suspected patients with COVID-19, accounting for 5.80%. 688 (85.79%) of 802 people who reported that they had taken effective personal protection measures for each exposure. Sex, work department, and length of service were the independent factors influencing the effective use of personal protective equipment in multivariate analysis (*P* < .05). **Conclusions:** Medical institutions need to continue to strengthen the training regarding standard precautions and personal protection, especially for general departments other than fever clinics and isolation wards, as well as medical staff with few working years, to ensure the occupational safety of medical staff.

**Funding:** No

**Disclosures:** None

